# Dielectrophoretic profiling of erythrocytes to study the impacts of metabolic stress, temperature, and storage duration utilizing a point-and-planar microdevice

**DOI:** 10.1038/s41598-023-44022-9

**Published:** 2023-10-12

**Authors:** Raphael Oladokun, Ezekiel O. Adekanmbi, Vanessa An, Isha Gangavaram, Soumya K. Srivastava

**Affiliations:** 1 Department of Chemical and Biomedical Engineering, West Virginia University, 1306 Evansdale Dr., PO Box 6102, Morgantown, WV 26506-6102 USA; 2 Summer 2022 High School Intern, Department of Chemical and Biomedical Engineering, West Virginia University, Morgantown, WV USA; 3 Present Address: Intel Corp, Chandler, AZ USA

**Keywords:** Biological techniques, Biophysics, Cell biology, Medical research, Engineering

## Abstract

Dielectrophoresis (DEP) is widely utilized for trapping and sorting various types of cells, including live and dead cells and healthy and infected cells. This article focuses on the dielectric characterization of erythrocytes (red blood cells or RBCs) by quantifying DEP crossover frequency using a novel point-and-planar microwell device platform. Numerical simulations using COMSOL Multiphysics software demonstrate that the distribution of the DEP force is influenced by factors such as the shape of the point electrode, spacing between the point and planar electrodes, and the type of bioparticle being investigated. The dependency on electrode spacing is experimentally evaluated by analyzing the DEP crossover response of erythrocytes. Furthermore, the results are validated against the traditional electrical characterization technique called electrorotation, which typically requires laborious fabrication and operation using quadrupole electrodes. Other significant factors, including erythrocyte storage age and the changes in cell properties over time since collection, osmolarity, and temperature, are also assessed to determine the optimal conditions for erythrocyte characterization. The findings indicate a significant difference between fresh and stored erythrocyte samples (up to 4 days), highlighting the importance of maintaining an isotonic medium for cell storage.

## Introduction

Research on red blood cells (RBCs or erythrocytes) holds significant importance in clinical hematology, such as transfusion medicine and anemia investigations, as well as in basic research including general membrane physiology or rheology^1^. The ability to transport and store a large inventory of human blood for transfusions is very important for medical institutions and research laboratories^2,3^. The study of RBCs employs various methodologies to explore their properties. Some causes of RBC artifacts or distorted attributes, which may arise when analyzing red blood cells (RBCs), include contamination due to poor RBC purification, temperature, shear stress, medium contents or supplements, and storage conditions^1^. These factors collectively contribute to changes in cell morphology and intracellular properties. Detecting these artifacts is important in scientific research and medical diagnostics to ensure the accuracy and reliability of results when working with RBCs. Subcellular components such as the lipid bilayer membrane, cytoskeleton, and cytoplasm are essential in maintaining cellular function and cell health. Environmental influences such as infections caused due to exposure to pathogens, and pharmacological interventions can impact these subcomponents' physiological and structural characteristics, influencing their biomechanical and bioelectrical properties^2^.

Various methodologies are employed to quantify the unique characteristics of RBCs. These include the use of radioactive tracers for measuring unidirectional ion flux^4,5^, patch-clamp techniques for electrophysiological studies of RBC cell membranes^6–8^, automated patch clamp for investigating the variability of RBCs among different donors, flow cytometry for counting and analyzing particles in the micrometer size range^1^, and cellular imaging for exploring cellular signaling^9^. Additionally, techniques such as holographic optical tweezers (HOT) and atomic force microscope-based single-cell force spectroscopy (SCFS) are employed for measuring adhesion force^10,11^. Dielectrophoresis (DEP), an electrokinetic technique, has proven to be more versatile for investigating RBCs' intracellular and membrane properties^12–17^. It estimates the electrical correlates of a cell's physiological state, including membrane conductance, capacitance, and cytoplasmic conductivity. These factors contain vital information about cellular function, ion transport across the membrane, and the propagation of electrical signals^18^. DEP revealed a critical function for dynamic potassium transport and casein kinase 1 activity when circadian rhythms were studied in red blood cells^19–21^. Other electric-based techniques used for biological cell manipulation include electrophoresis, electroosmosis, electrothermal, and dielectrophoresis of red blood cells in direct-current (DC)^22^ and alternating-current (AC)^3,23,24^ devices.

Over the years, dielectrophoresis has developed into a powerful, efficient, and flexible method as a basic cell research tool for cell characterization, manipulation, separation, and biological cell patterning. It has been employed to investigate various cells using AC and DC electric fields^15,17,25–29^. The characterization of dielectric properties of biological cells is a crucial step towards the numerical scanning of cell behavior under external electric fields^30–34^, which can aid in the design of diagnostic tools through DEP)^16^. Cells are typically suspended between electrodes, and their behavior is observed when external electric signals are applied^35–38^. The spatial arrangement of these electrodes usually generates a nonuniform electric field when they are functionalized with the electric signals. This nonuniform electric field causes cells that are suspended in the medium^36,37,39^ to either move towards the low-field region (negative DEP, i.e., nDEP) or high-field region (positive DEP, i.e., pDEP)^14^. The AC frequency at which no cell movement is observed, i.e., when cells tend to transit from negative to positive DEP or vice versa, is termed the "crossover frequency," which can provide critical information about the cells’ dielectric properties^30,40^. Pohl and Hawk carried out the first application of DEP to separate live and dead cells in 1966. Since then, this technique has been utilized in various biological systems and components, including bacteria, yeasts, stem cells, viruses, mitochondria, and DNA^15,16,41^.

The crossover frequency refers to a specific frequency at which suspended particles experience a transition in their behavior to electric field due to DEP forces. At this frequency, the DEP force approaches zero, shifting from attraction to repulsion or vice versa. It pulls the suspended particles towards or pushes them away from high-field regions, usually around the electrode region. Lavi et al., in a recent study, stated that the resulting frequency-dependent DEP force curve, or DEP spectrum, will consist of finite field frequency ranges where cells are either attracted towards (positive DEP) or repelled away from (negative DEP) the high electric field regions as defined by the array's electrodes. The frequency in which this attraction/repulsion reverses and cell DEP motion ceases is defined as the DEP crossover frequency (*f*_*CO*_) ^2^. The crossover frequency depends on the size and shape of the suspended particles, membrane morphology, cytoplasm components, and the characteristics of the suspending medium and the electric field applied^29,35,42–45^. The most common DEP applications of crossover frequency include particle enrichment, separation, and characterization, which are utilized for various biomedical, biotechnological, and analytical purposes.

Various electrode configurations, including polynomial, interdigitated, strip, U and T, needle, and many more, have been used to generate a nonuniform electric field for determining the electrical properties of bioparticles. These properties have been studied for various bioparticles, such as bacteria^46^, fungi^47^, cancer cells^48^, human red blood cells^49^, and others^14,37^. However, the spacing, shape, and electric potential differences between these electrodes can impact the distribution of the electric field within the cell characterization device platform. Designing and fabricating such electrode setups can be expensive and cumbersome. These electrode configurations typically consist of fixed metal-based electrodes, which restrict the real-time exploration of cell behavioral effects at different inter-electrode distances. The fixed electrode spacing may limit the dynamic investigation of bioparticle behavior. However, in other characterization platforms, a single measurement can take up to 30 min, resulting in electrode degeneration after multiple experimental runs^50^ and the loss of viability of suspended bioparticles before the completion of experiments. Furthermore, using multiple platforms can introduce device variability, leading to measurement errors, unless statistical verification confirms the insignificance of this variability. However, employing various devices using current state-of-the-art characterization methods can be costly, even with statistical evidence.

This paper presents a simple and novel point-and-planar electrode microwell (PPM) for dielectric characterization of bioparticles based on the principles of dielectrophoresis. The fabrication process of the PPM, which utilizes a silicone-based polymer and high-grade platinum electrodes, is discussed. The numerical investigation of inter-electrode spacing and electrode shape's influence on DEP force is analyzed using a commercial software package, COMSOL Multiphysics v5.3a, and the findings are experimentally verified using red blood cells. The same device is also used to quantify the effects of cell storage period, osmotic stress on their membrane capacitance, and the impact of temperature on the crossover frequency and membrane capacitance. The entire experimental setup is compared to an alternative technique, electrorotation (ROT)^51^.

## Materials and methods

### Point-and-planar microwell (PPM) device architecture and experimental setup

**Figure 1:**
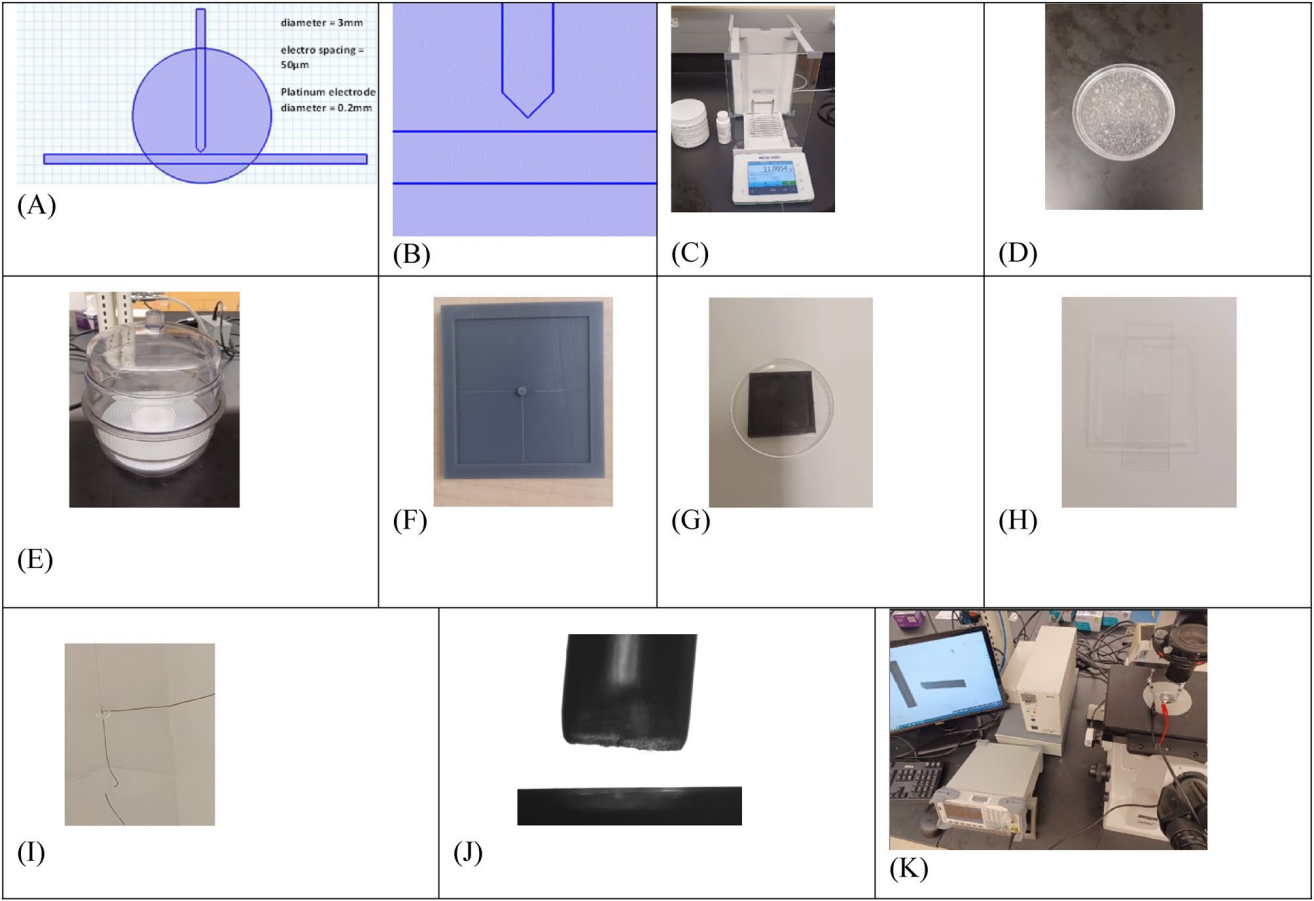
Design and fabrication of a Point and Planar Electrode Microwell (PPM) Platform and experimental setup for DEP analysis. (**A**, **B**) PPM platform design at 50 μm electrode spacing. (**C**–**K**) Fabrication process and experimental setup for Point and Planar microwell device for DEP analysis. (**C**, **D**) Measure and mix the elastomer and curing agent in a 10:1 ratio. (**E**) Degass the mixture. (**F**, **G**) PDMS mold obtained by 3D printing. (**H**, **I**) Sealing the PDMS using plasma wand and assembling the electrodes. (**J**) Observe the electrode geometry under a microscope. (**K**) Experimental set up that includes a signal generator (having sine waveform), inverted microscope, desktop computer, and Teledyne video camera.

The PPM device comprises two high-quality platinum electrodes, with a variable-height perforated polydimethylsiloxane (PDMS) layer sandwiched between two 1 mm thick borosilicate glass plates (Fig. 1). The microwell in the PDMS layer has a diameter of ~3 mm to ensure rapid equilibration of the bioparticle suspension. The planar electrode is fixed, while the point electrode's position in space can be varied using the stage and ocular micrometer to measure the distance between the electrodes accurately. This variable inter-electrode distance enables exploration of the bioparticle’s response dependence on the electric field strength at a fixed voltage for both nDEP and pDEP. A large inter-electrode spacing would prolong the bioparticle's residence time in the nDEP or pDEP domain, which may alter the cells' integrity, leading to inaccurate measurements. Conversely, if the electrode spacing is too small, some bioparticles may experience strong pDEP, increasing the lysis of cells, biofouling of the electrodes, and ultimately degrading the electrodes. Optimum electrode spacing can help prevent bioparticle lysis or electrolysis at the electrodes within the PPM characterization platform.

PPM has several advantages, including cost-effectiveness, as it uses a simple electrode design and PDMS polymer for fabrication. Additionally, PPM offers the flexibility of being constructed with considerable height, which improves optical clarity during experimentation with bioparticles of different sizes. Another advantage of PPM is its quick response to the electric field, taking < 2 min. to transition from nDEP to pDEP and vice versa. The steps required for fabricating the microwell system are shown in Fig. 1 and will be explained in the next section.

### Device fabrication

Electrokinetics, a branch of physics studying the motion of neutral or charged particles under electric currents, involves using various materials to contain bioparticles for a range of analyses. These materials include glass^52,53^, ceramics^54–56^, silicon^57,58^, polymer^59–61^, composites^62,63^, hydrogel^64,65^, and paper^66,67^. For microfluidic applications, commonly used materials include thermoplastic polymers such as polycarbonate^68^, polyethylene glycol diacrylate (PEGDA)^69^, poly-methyl methacrylate (PMMA)^70^, fluorinated ethylene propylene (Teflon FEP)^71,72^, polyurethane (PU)^73^, polystyrene^74^, elastomer PDMS^75,76^, or thermoset polyester (TPE)^77,78^.

PDMS was chosen as the material for the PPM platform due to its unique properties, such as non-toxicity, high transparency, deformability, gas permeability, low auto-fluorescence, and low cost^79^. PDMS can also be cured with moderate heat or under ambient conditions without an ultraviolet or argon light source. This PDMS PPM platform is complemented with borosilicate glass to enhance sealing, hydrophilicity, and visibility under the microscope. Sylgard 184 Silicone Elastomer (Dow Corning, Midland, MI, USA) was mixed with its curing agent and cured as described previously^61^. Briefly, Sylgard 184 silicone elastomer base and curing agent in the ratio 10:1 were mixed. The mixture was degassed, resulting in an optically clear slurry, thermally cured at 70 °C for 90 min. in a Blue-M oven, as shown in Fig. 1^80^. The cured elastomer was cut into 50 × 50 mm^2^ squares. Microwells of 3 mm diameter were created in the PDMS squares to house a pair of perpendicularly arranged, 0.008" diameter, 99.95% pure platinum wires that were exposed to plasma using an in-house microwave plasma cleaner^81 ^ or commercially available plasma wand and then sealed onto a 0.5 mm thick borosilicate glass slide. High-grade 0.008" diameter platinum wires were arranged as shown in Fig. 1. The distance between them was set using an Olympus IX inverted microscope. One electrode served as a point electrode, while the other was the planar electrode. Once electrodes were positioned using the microscope, Loctite® epoxy mixture was used to permanently adhere the electrodes into position so that the spacing between electrodes were fixed.

### Device sealing

An in-house microwave generator^82^ or a commercially available plasma wand is used for sealing. The plasma treatment modifies the alkyl group's interaction on the polymer surface with the glass and changes them chemically to a hydroxyl (–OH) functional group. This results in a hydrophilic microwell with a lower surface energy and contact angle. Thus, it prevents the formation of medium-air convex interfaces (relative to air), which can interfere with the optics and spatial distribution of bioparticles.

### RBC sample preparation

In this study, bovine erythrocytes (C-95053, Washington State University, USA) were obtained and subjected to two washes in phosphate-buffered saline (PBS) with a pH of 7.2. Following the washes, the erythrocytes were suspended in an isotonic dextrose medium with a conductivity of 0.056 S/m. The experiment was subsequently replicated at different medium conductivities by adjusting the conductivity using potassium chloride (KCl).

To study metabolic stress on the cells, the medium's osmolality was adjusted to be hypotonic, isotonic, or hypertonic by preparing three sets of media with different concentrations of sodium chloride (NaCl): 0.45% NaCl solution (hypotonic), 0.9% NaCl solution (isotonic), and 2% NaCl solution (hypertonic). Freshly collected bovine erythrocytes were suspended in each of the three NaCl solutions. The cells were used within four hours after being suspended in a hypertonic and hypertonic solution, as the rate of cell swelling is time dependent.

As described by Gascoyne et al*.*^51^, human red blood cells were used for comparison. Packed erythrocyte fractions from a group-O blood sample (Research Innovation Incorporated, VA, USA) were washed twice in PBS (pH 7.2) and then suspended in a medium containing 8.5% (w/v) sucrose and 0.3% (w/v) dextrose of conductivity 52 mS/m. The medium conductivity was adjusted by adding potassium chloride salt (KCl), and the experiment was repeated to obtain DEP crossover frequency. This process was repeated for seven medium conductivity values ranging from 0 to 60 mS/m.

### Statistical analysis

A paired *t* test (two-tailed) was conducted on the crossover frequency measurements for each of the seven conductivities. The experiment followed a 3 × 4 factorial design, with two factors: electrode shape and spacing at 3 and 4 levels, respectively. Two-way ANOVA (GraphPad Prism version 8.4.3 for Mac, GraphPad Software, San Diego, California, USA) was used for statistical analysis to determine the interaction effects between the categorical variables (electrode spacing and shape) and the continuous dependent variable (crossover frequency), as well as to determine the main effects of each factor and their interactions.

## Theory

Maxwell–Wagner (MW) interfacial polarization mechanism is essential for designing and optimizing various electronic devices, energy storage systems, and sensor technologies. DEP is a phenomenon that defines the field-induced force applied to a polarizable particle in a nonuniform electric field, which arises from MW interfacial polarization^83^. Nonlinear electrokinetic stress (which scales as the square of the voltage) acts on the induced dipole moment at the surface of a particle. Nonlinear electrokinetic effects are responsible for dielectrophoresis, electroosmosis, electrothermal flows, and induced charge electrokinetics. Nonlinear electrokinetics is caused by the interaction between the imposed electric field and the electrical charge induced by the electric field itself, typically following a dipolar pattern. In contrast, linear electrokinetics originates from the action of the imposed electric field on fixed charges, including both interfacial fixed charge and fixed diffused charge within the native electric double layer (EDL). Both interfacial fixed charge and fixed diffused charge are components of the EDL that form at the interface between a charged surface and an electrolyte solution. The PPM device platform works on the principle of DEP crossover frequency measurement^43,84–87^. The DEP force and the Clausius Mossotti factor ${f}_{CM}$, i.e., Eqs. 1 and 2 guide the operation of PPM. The Clausius Mossotti factor describes the electrical polarization of the cell in its suspending medium. Whenever two equal but parallel plates with the voltage of opposite signs (positive and negative) are separated by a predetermined distance, an electric field is produced between them. Healthy erythrocytes have been modeled using an oblate spheroid model, but as explained previously, this spherical approximation is appropriate for parasitized cell populations where considerable cell geometry variations occur^83^. The polarizability factor, ${f}_{CM}$, of cells depends on their specific membrane capacitance, membrane conductivity, and the internal permittivity and conductivity^83^. The Clausius–Mossotti factor for a spherical particle (homogeneous lossy dielectric sphere) given by Eq. 1, approximates the electrical polarization of the cell with respect to its suspending medium^83^, *represents a complex entity, $\sigma$ the conductivity, $\varepsilon$ the permittivity, and $\omega$ the field frequency, respectively. Equation.1 reveals that permittivity dominates particle polarization at high frequencies, while conductivity is the governing factor at low frequencies^88^.

1 $${f}_{CM}=\frac{{\varepsilon }_{cell}^{*}-{\varepsilon }_{medium}^{*}}{{\varepsilon }_{cell}^{*}+2{\varepsilon }_{medium}^{*}} \;where \;{\varepsilon }_{k}^{*}={\varepsilon }_{k}-\frac{i{\sigma }_{k}}{\omega }; k=cell,medium$$

The real components of ${f}_{CM}$, Re(${f}_{CM}$), coupled with the spatial inhomogeneity and traveling components of the applied electrical field, create the necessary DEP force^72^. These forces depend not only on the electrode array's geometrical configuration and excitation scheme but also on the dielectric properties of the bio particle and its suspending medium. The DEP force acting on the particle can be estimated by Eq. 2.

2 $${F}_{DEP}=4\pi {r}^{3}{\varepsilon }_{m}Re\left({f}_{CM}\right)\left|{E}^{2}\right|\approx Re\left({f}_{CM}\right){V}^{2}$$

where ${F}_{DEP}$ is the dielectrophoretic force; *r* the particle radius; ${\varepsilon }_{0}$ the permittivity of the vacuum; ${\varepsilon }_{m}$ the permittivity of the suspending medium; E the electric field, $Re\left({f}_{CM}\right)$ is the real part of the Clausius–Mossotti factor, and V is the voltage^71^. A positive $Re\left({f}_{CM}\right)$ signifies that the particles are drawn towards the electrodes (pDEP), while a negative $Re\left({f}_{CM}\right)$ implies that particles are repelled from the electrodes (nDEP).

**Figure 2:**
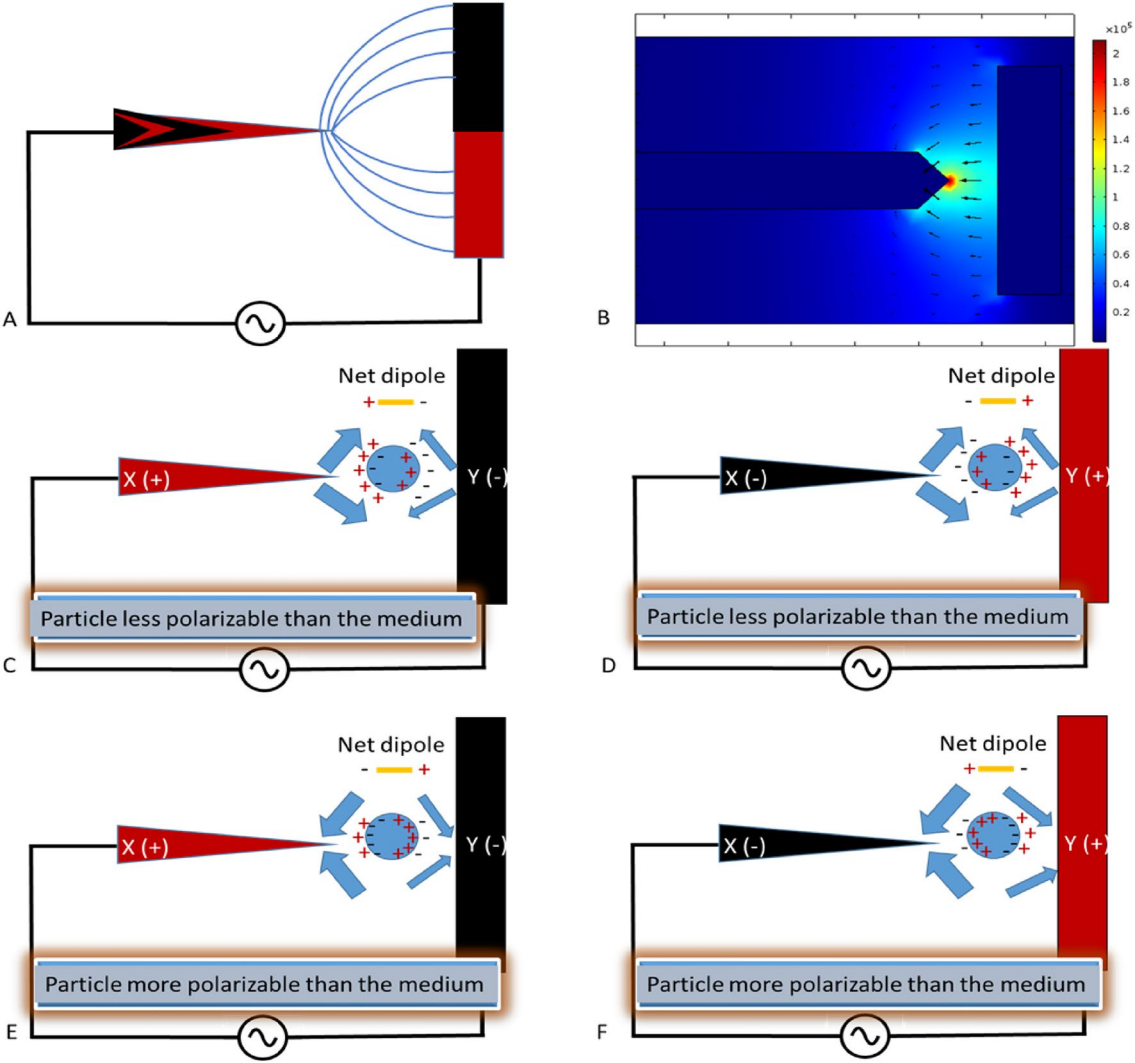
Nonuniform field effects on neutral but polarizable bioparticles. (**A**) nonuniform field lines representing the unequal field strength are numerically demonstrated. (**B**) COMSOL simulation of PPM functionalized with 8 V_pp_, 100 kHz AC signal. (**C**) shows neutral particles experiencing nDEP force. (**D**) When the charge on the electrode in (**C**) is switched, the same nDEP effect is observed. (**E**) Here, the particle is more polarizable than the medium, and pDEP is in effect. (**F**) Switching the polarity of the electrodes in (**E**) resulted in the same pDEP effect observed by the bioparticle. Hence, there is no dependency on electrode polarity, and the response of cells will be the same. The bigger arrows indicate the dominant dielectrophoretic force and the cell response.

Depending on the nature of the particle, a torque may be generated (if the particle is elongated or has unequal polarizability) or not (if the particle is spherical or isotropic in its polarizability), even though there is no net translational movement^89^. With the point-and-planar electrode arrangement (Fig. 2), the electric field is rendered nonuniform in agreement with the principle of DEP. However, because the dielectrophoretic force relies not only on the properties of the bioparticles but also on the particle's suspending medium, Fig. 2 will be explained in terms of both the particle and the suspending medium characteristics.

Assuming a neutral (polarizable) bioparticle is suspended in a highly conductive medium, both the particle and the medium will be polarized under the influence of an external electric field. At any moment within the AC field between electrodes X and Y, X is positively charged, and Y is negatively charged. Therefore, the neutral particle suspended between electrodes X and Y will be polarized, as shown in Fig. 2C. Because the medium is highly conductive, counterions will accumulate at the interface between the particle and the medium, resulting in a net dipole, as depicted in Fig. 2C. In the nonuniform electric field established between electrodes X and Y, the electric field strength is higher at X than at Y (Fig. 2B).

With the medium more polarizable than the particle and the electric field stronger at X, the negative-negative repulsive force at X is greater than the positive-positive repulsive force at Y, pushing the particle away from X towards Y, causing nDEP. If the electrode polarity is reversed, the same nDEP effect on bioparticles will be observed (Fig. 2D). When the opposite happens, and the particle is more polarizable than the medium (Fig. 2E), there is a switch in the net dipole orientation, and the attractive force by the anode (X) on the dipole is greater than that of the cathode (Y). As a result, the dipole is pulled towards X, causing pDEP. If the electrode polarity is reversed, such that X is the cathode and Y is the anode (Fig. 2F), the same pDEP effect will be observed since the orientation of the dipole depends on the electrode polarity. When the particle and the medium have equal polarizability, there will be no net movement of the particle. In other words, the Clausius–Mossotti factor is zero, and the particle appears unresponsive to the external field effects, known as the crossover frequency.

## Results and discussion

### Effect of point electrode shape and inter-electrode spacing

The spacing between the two Pt electrodes determines the magnitude of the electric field and DEP force between them. Additionally, the shape of the point electrode (as shown in Fig. 2) defines the spatial distribution of the electric field. It is important to note that the spacing between the electrodes determines which of the two terms of the time-averaged DEP force acting on a spherical particle is dominant. The dipole force term dominates the overall DEP force for particles with diameters less than one-tenth of the inter-electrode spacing. For particles larger than this, the quadrupole force term becomes more prominent^75^. The time-averaged DEP force acting on a spherical particle can be calculated by considering both the dipole and quadrupole terms as described by Washizu^76^.

$${F}_{DEP}=\mathrm{dipole\, force\, term }+\mathrm{ quadrupole \,force \,term}$$

3 $$F_{DEP} = 2\pi r^{3} \varepsilon_{m} Re\left[ {\frac{{\varepsilon_{cell}^{*} - \varepsilon_{medium}^{*} }}{{\varepsilon_{cell}^{*} + 2\varepsilon_{medium}^{*} }}} \right]\left| {E^{2} } \right| + \frac{2}{3}\pi r^{5} \varepsilon_{m} Re\left[ {\frac{{\varepsilon_{cell}^{*} - \varepsilon_{medium}^{*} }}{{\varepsilon_{cell}^{*} + 2\varepsilon_{medium}^{*} }}} \right]\left| {E^{2} } \right|$$

A substantial change in crossover frequency occasioned by a shift in the nDEP and pDEP domain will affect the estimated dielectric properties, i.e., capacitance and permittivity of the particle. The effect of electrode spacing is numerically analyzed on the DEP factor (i.e., the magnitude of the dot product of the electric field, |E. E|) that will influence the dielectrophoretic force experienced by any polarizable particle suspended between the electrodes. Equation.3 represents the resulting DEP force^90^. With vector transformation on the electric field, as shown by Pethig, in reference to Hsu et al.^91^ gives:

4 $$2\left(E.\nabla \right)E=\nabla \left(E.E\right)-\left(E.\nabla \right)E-E\times \left(\nabla \times E\right)-(E\times \left(\nabla \times E\right)=\nabla (E.E)$$

leading to:

5 $${F}_{DEP}=2\pi {r}^{3}{\varepsilon }_{0}{\varepsilon }_{m}Re\left[CM\right]\nabla (E.E)$$

Hence, the knowledge of |E. E| indicates the variation in the dielectrophoretic force within the microwell. The Clausius–Mossotti factor close to the DEP crossover frequency range is given by^75^:

6 $$Re\left[CM\left(f\right)\right]\approx \frac{{f}^{2}-{f}_{xo1}^{2}}{{f}^{2}+{2f}_{xo1}^{2}}$$

substituting $Re\left[CM\left(f\right)\right]$ in Eq. 6 gives the time–average DEP force of a spherical, viable cell, and this can be expressed as:

7 $${F}_{DEP}=2\pi {r}^{3}{\varepsilon }_{0}{\varepsilon }_{m}\frac{{f}^{2}-{f}_{xo1}^{2}}{{f}^{2}+{2f}_{xo1}^{2}}\nabla (E.E)$$

Since the conductivity of the medium and the particle geometry can be measured directly, the determination of the first crossover frequency *f*_*x01*_ can be used to estimate the capacitance of the plasma membrane^75^.

8 $${f}_{xo1}\approx \frac{1}{\sqrt{2}} \frac{{\sigma }_{med}}{\pi r{C}_{mem}}$$

The numerical approach used to investigate the effect of electrode spacing involved designing three different shapes for the point electrode while fixing the planar electrode (Fig. 5). The electrodes were charged with 8 V_pp_ (± 4 V, i.e., peak-to-peak voltage) to establish the distribution (gradient) of the electric field. Simultaneously, the Laplace equation ∇^2^φ = 0 was solved in stationary mode using the AC/DC module in COMSOL Multiphysics software v5.3. The Laplace equation was solved without relying on either Navier–Stokes (for the transport of momentum) or the convection–diffusion equation (for the transport of mass) to focus solely on the effects of the electric field. Details on using the Laplace equation to explore the distribution of electric field effects have been reported previously^92^. Different electrode shapes were employed to allow the region of high field strength to be spatially identified, aiding in the pinning-down of both pDEP and nDEP regions during experiments. Computing the spatial gradients of the electric field is crucial for bioparticle characterization, enrichment, and sorting.

**Figure 3:**
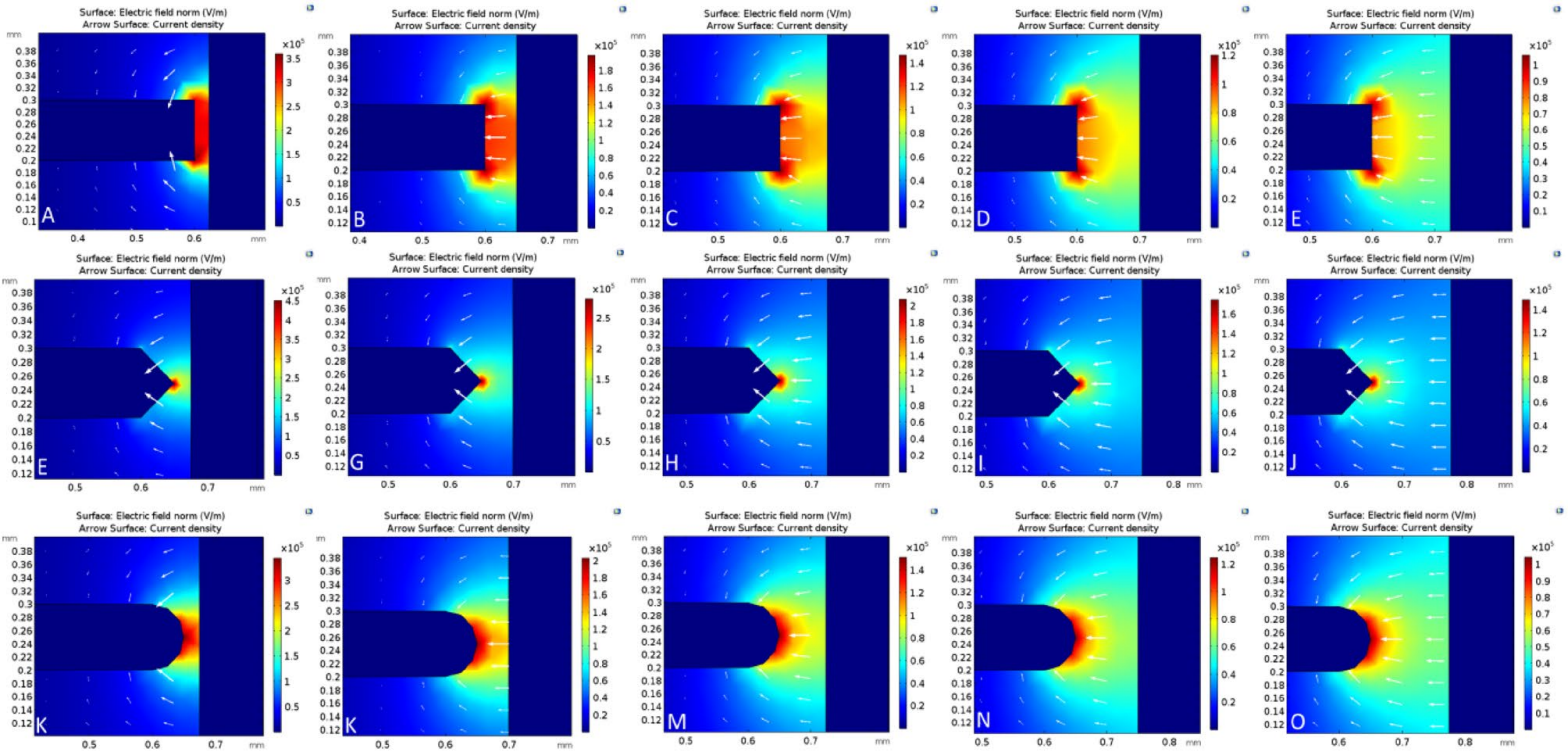
Electrode shape and inter-electrode spacing effects on the electric field norm and current density. (**A**–**E**) (top row), (**F**–**J**) (middle row), and (**K**–**O**) (bottom row) consist of progressively varying inter-electrode distances, i.e., 25 µm, 50 µm, 75 µm, 100 µm, and 125 µm for three different point-electrode shapes: square (top row), triangular (middle row), and semicircular (bottom row). The triangular-shaped design has the highest field strength, while the semicircular has the least.

As shown in Fig. 3, the electric field distribution varies for different electrode spacings and shapes of the point electrode. As the distance between the electrodes increases from 25 to 125 µm, the maximum electric field strength decreases across the three designs. For instance, in the triangular design (Fig. 3F–J), the maximum field strengths at 25 µm and 125 µm are 4.5 * 10^5^ V/m and 1.5 * 10^5^ V/m, respectively. Also, the maximum field strength at 25 µm for a square-shaped electrode is 3.6 * 10^5^ V/m and 1.05 * 10^5^ V/m for the semicircular design. At 125 µm, their field strengths are similar, at 1.04 * 10^5^ V/m for the square and semicircular electrodes. This shows that the maximum field strength among the designs at a fixed inter-electrode spacing is ordered as follows: −∇V_triangle_ > −∇V_square_ ≥ −∇V_semicircle_. However, at a fixed inter-electrode distance of 125 µm, the maximum field strength of 1.5 * 10^5^ V/m was the highest observed for the triangular shape but remained almost the same for square or semicircular shapes. Furthermore, the effect of dielectrophoretic force on these electrode shapes and spacings is explored when a polarizable particle is suspended between the electrodes.

A cut-plane tool was used to define a one-dimensional path along the horizontal axis so that the end of the point electrode is symmetrically connected to the planar electrode. This is commonly defined as the arc length (Fig. 3F–H). As mentioned in Eq. 4, its absolute values were tracked along the defined arc length and plotted against the normalized arc length by numerically calculating the DEP factor. Figure 3 A-E shows the variation of the DEP factor at each of the inter-electrode spacings for the three designs. At the lowest spacing (25 µm), the triangular and semicircular designs show a progressive decrease in the DEP factor from point Y to X, unlike the square pattern that initially shows a progressive increase up to 80% of the arc length before changing course toward the downward trend. The square-shaped electrode displayed a similar trend when other spacings were analyzed, i.e., 50, 75, 100, and 125 µm. In contrast, other shapes maintained their trend, even though there was a progressive decrease in the DEP force experienced by the bioparticle.

**Figure 4:**
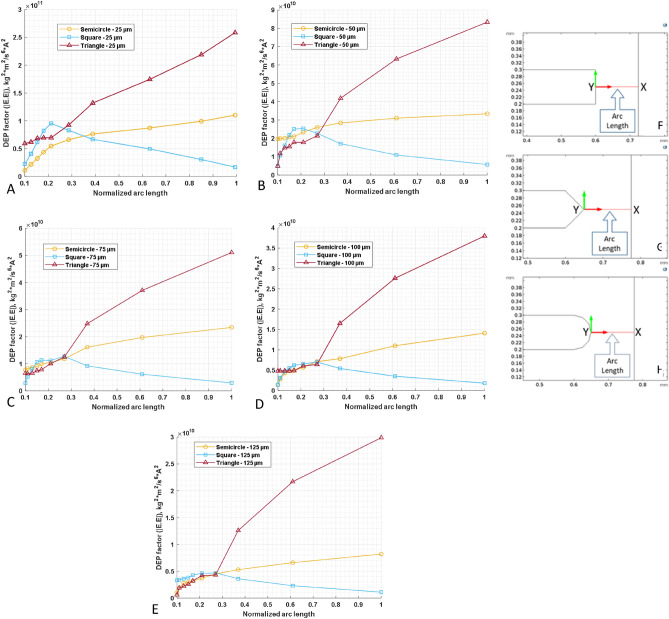
The plot of the variation of the DEP factor along the arc length. (**A**–**E**) represents the variation in DEP factor for 25–125 µm spacing. (**F**–**H**) are the arc lengths for square, triangular, and semicircular electrode shapes, respectively, with X being the low end and Y the high end of the DEP factor.

**Figure 5:**
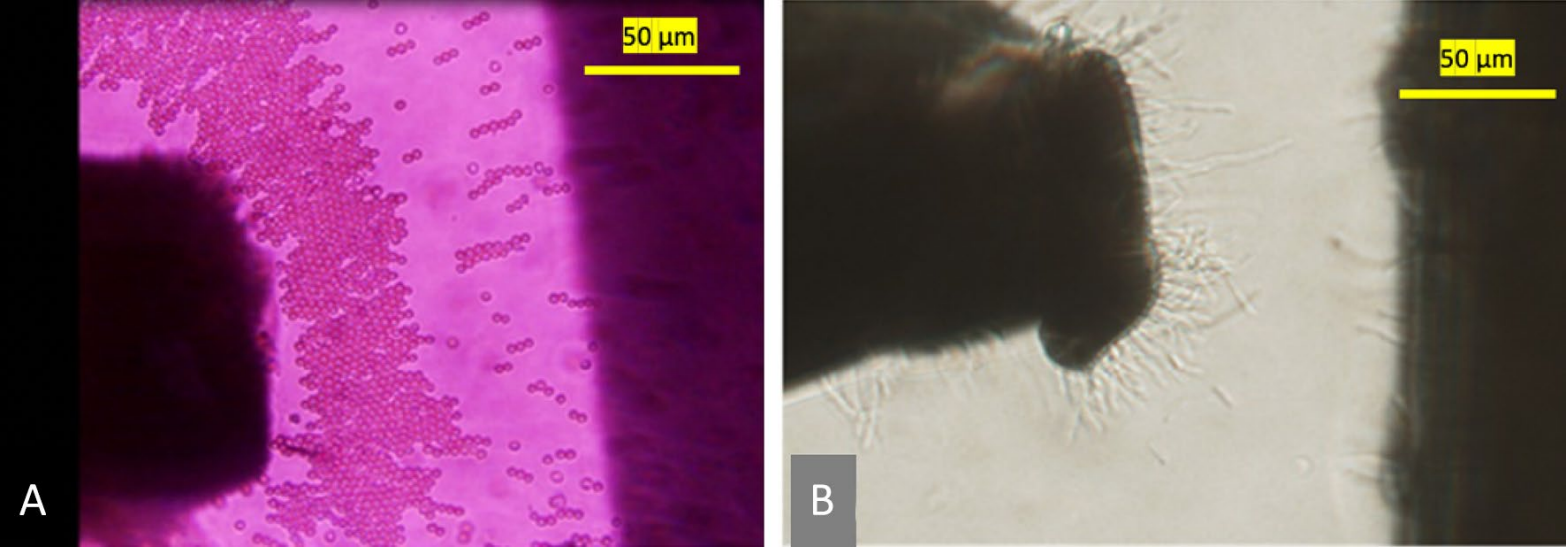
The pre-experimental PPM device platform setup for square (**A**) and pseudo-triangular (**B**) shaped electrodes at 100 µm and 75 µm inter-electrode spacing, respectively. Also, (**A**) and (**B**) are bovine red blood cells and *Cupriavidus necator* bacteria experiencing pDEP as eukaryotes and prokaryotes, respectively. Cells in (**A**) are farther apart from the point electrode than (**B**) that are binding to the surface of the triangular electrode, validating simulations in Fig. 4.

The physical significance of the simulation presented in Fig. 4 is that bioparticles are expected to cluster around the Y region when experiencing pDEP for both semicircular and triangular electrodes. However, based on Fig. 4, for a square-shaped electrode, bioparticles will not cluster at Y when experiencing pDEP; rather, they will only cluster close to Y depending on the inter-electrode spacing. Figure 4 demonstrates that irrespective of the electrode spacing and, apart from the square-shaped electrode, the other two shapes, i.e., triangle and semicircle, would have their highest DEP factor at Y. This observation was tested experimentally for both square (Fig. 5A) and triangle (Fig. 5B) shapes using prokaryotic and eukaryotic cells. Using these two cell types shows the versatility of the point-and-planar microwell device platform. Figure 5A shows bovine red blood cells (RBCs) experiencing pDEP at 800 kHz, 8 V_pp_, and not adhering entirely to the point electrode. This is because the highest DEP factor, according to Fig. 4, is not at the point electrode (Y) for the square-shaped electrode but somewhere between the two point-and-planar electrodes. Further experimental validation was conducted using bacteria, as shown in Fig. 5B. As seen in Fig. 5B, the cells were perfectly adhered to the surface of the point electrode during the pDEP regime at frequencies ≥ 300 kHz and 8 V_pp_. The experimental observations in Fig. 5 validates the simulation data presented in Fig. 4.

**Figure 6:**
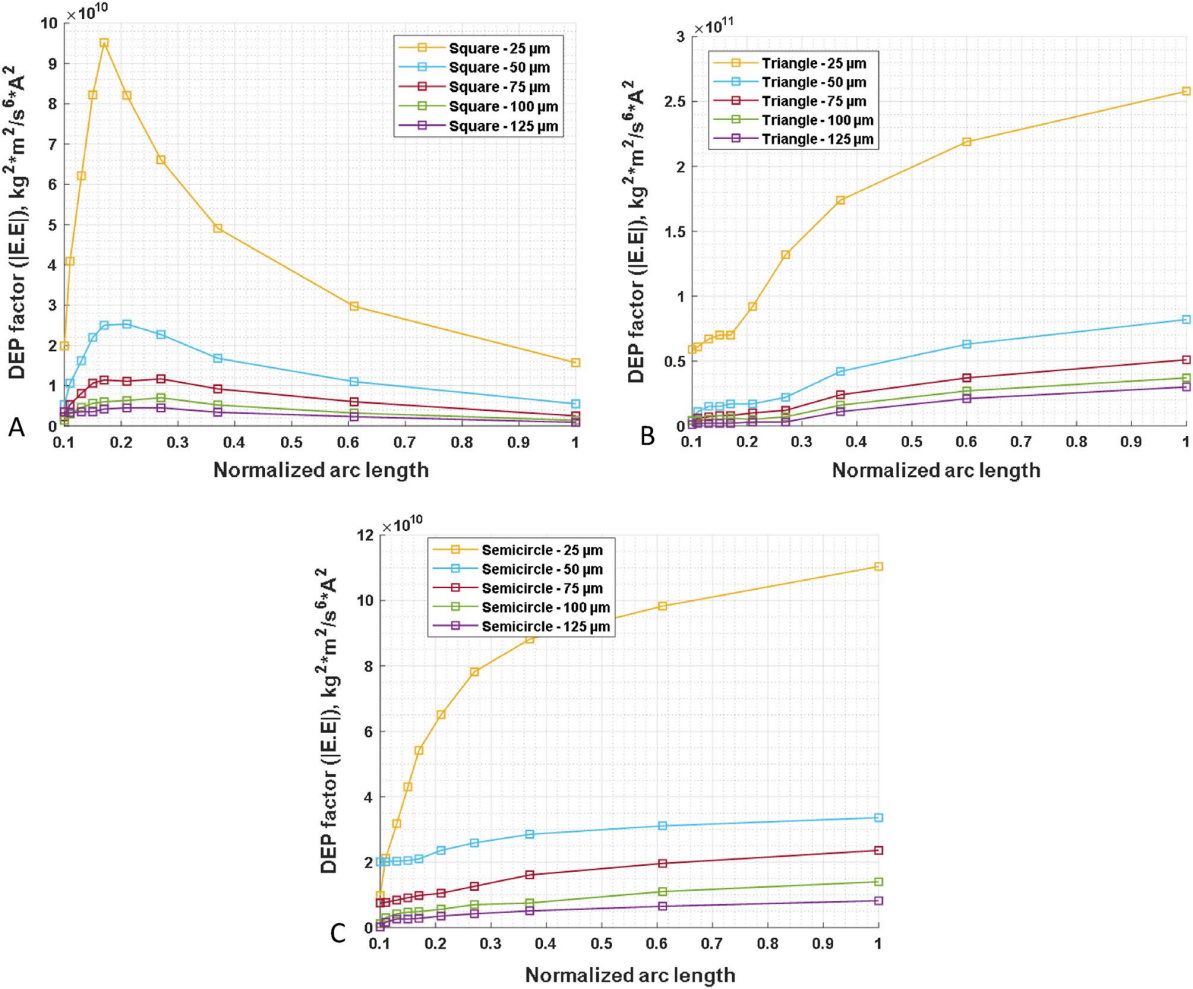
Plots (**A**–**C**) combine the variation of the DEP factor for each shape: square, triangle, and semicircle-shaped electrodes, respectively. At a spacing of 25 µm, the DEP factor is distinctly higher than all other spacings explored, irrespective of the shape.

When plotting the DEP force factor for each shape (seen in Fig. 6), the dielectrophoretic trend for each shape became more pronounced. The triangle shape was consistent with respect to spacing, as seen in Fig. 6B. Additionally, the DEP factor for the triangle shape is highest at a 25 µm electrode spacing compared to the other shapes. The square shape has a parabolic-shaped trend, as seen in Fig. 6A. The square shape maintains its hilly nature with a gradual shift in the peak value from left to right. Experimentally, regardless of the electrode shape, utilizing bovine RBCs at 8 V_pp_ and a spacing of 25 µm always resulted in electrolysis. However, the observed cell movement was too slow at a spacing of 125 µm, irrespective of the electrode shape. Therefore, a workable inter-electrode distance range for the PPM device platform was between 50 and 100 µm spacing.

### Impact of cell storage age on membrane capacitance

The effects of cell storage period on cell properties and their influence on membrane capacitance were investigated in this study. It was observed that as cells were stored for longer durations since collection, changes in their properties occurred, resulting in variations in membrane capacitance. In direct current (frequency-independent) dielectrophoresis (DC DEP), the separation of cells into individual components heavily relies on membrane capacitance, which influences membrane conductance. If the dielectric characterization results are erroneous, then the design and testing of the microfluidic platform will be flawed, as the obtained membrane capacitance is utilized in the modeling and simulation of the sorting device platform. One of the factors that can affect this dielectric property characterization is the storage period of the cells, i.e., how fresh the sample is once drawn from animals or humans. This also means the length of time the RBCs are stored in a 4 °C refrigerator before being utilized for experiments. Many molecular and clinical reports have provided compelling evidence that the long-term storage of erythrocytes will likely decrease their quality^93–98^. Jeon et al*.* studied the changes in properties when RBCs were stored for 40 days using optical tweezers^49^. A dramatic change in the DEP response of the cells was observed as the cells aged. Here, the PPM platform will be used to investigate the effect of cell storage period on membrane capacitance at 4 °C storage conditions for four (4) days. For this study, we considered the heterogeneity of the cells for nine different samples, ignoring electrode shape effects since there was not much change in the crossover frequency observed.

**Figure 7:**
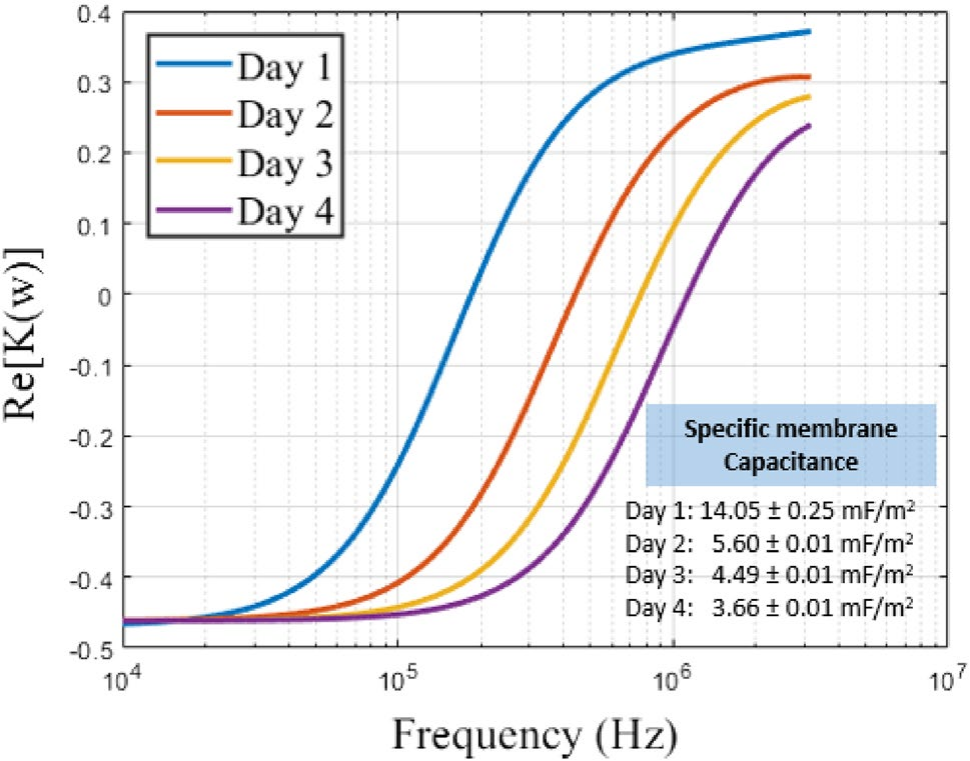
The plot shows the variation of the real part of the Clausius–Mossotti factor as a function of frequency. The intersection point of the sigmoidal curve and the horizontal line represents the crossover frequency of the cells. The increase in storage period resulted in cells having a higher crossover frequency due to cell shrinkage, validating the high crossover frequencies obtained for smaller particles.

When the crossover frequency data were analyzed and plotted using the theoretical single-shell model, a substantial shift in the bovine RBC properties correlating to the cell DEP response from days 1 – 4 was observed, as shown in Fig. 7. This shift can be related to changes in the cell membrane as the cell ages. As the days go by, the ability of the cell membrane to resist electric fields (permittivity) weakens due to the degradation of the membrane, as the membrane permittivity is directly proportional to the membrane capacitance. The average membrane capacitance values obtained are shown in Fig. 7 for a sample size of n = 3. This decrease in permittivity (associated with increased crossover frequency) is consistent with the reported variation in DEP force using the optical tweezer technique^49^. Biophysically, this could be correlated to the size of the cells, i.e., the cells shrink, reducing the membrane area and thus leading to a decrease in capacitance as storage period increases.

**Figure 8:**
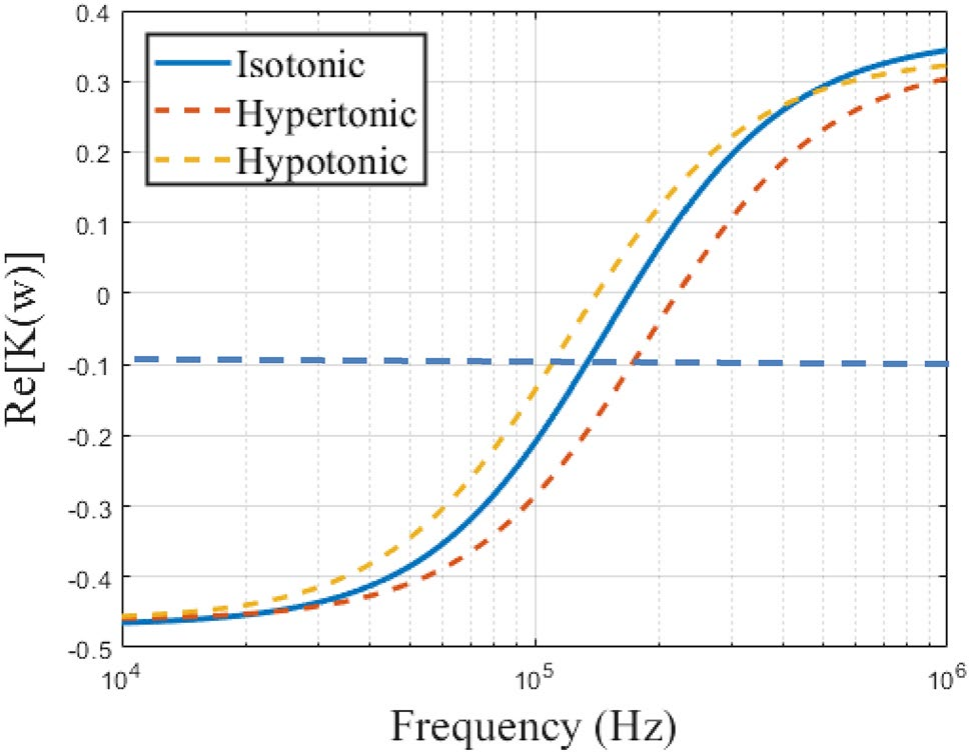
The plot of the variation of the real part of the Clausius–Mossotti factor as a function of frequency. The point at which the sigmoidal curve intersects the horizontal line is the crossover frequency of the cells. The hypertonic solution resulted in cells having higher crossover frequency due to the cell shrinkage, validating the large crossover frequencies obtained for smaller particles. Each data point was acquired with three independent samples and six repetitions of each sample at varying medium conductivity.

Based on the simulation shown in Fig. 6, there is no significant variation in the electric field strength among different electrode shapes. A rectangular-shaped electrode was used for this study, and the normalized experimental results are reported in Figs. 7 and 8.

### Effects of dynamic stress on membrane capacitance

Another factor that could affect the outcome of the dielectric characterization of any biological cell is the osmolality of the suspending medium. The crossover frequency for each set of RBC samples (n = 3) was determined at varying suspending medium conditions (isotonic, hypotonic, and hypertonic), and a single-shell model was used to obtain the membrane capacitance, as shown in Fig. 8. From Fig. 8, it is evident that the crossover frequency of bovine RBCs was affected by the suspending medium's osmolality. In the hypotonic solution, the RBCs' size slightly increased due to turgidity, resulting in a lower crossover frequency, as reflected in Fig. 8. An isotonic solution is preferred because it provides a realistic data of the cell membrane capacitance since the membrane is not compromised through water loss or gain. When the hypertonic medium was used to suspend the bovine RBCs, the crossover frequency increased due to the reduction in cell size. These results confirm the importance of maintaining a specific osmolality when characterizing biological cells.

### Effects of temperature on the cell membrane capacitance

**Figure 9:**
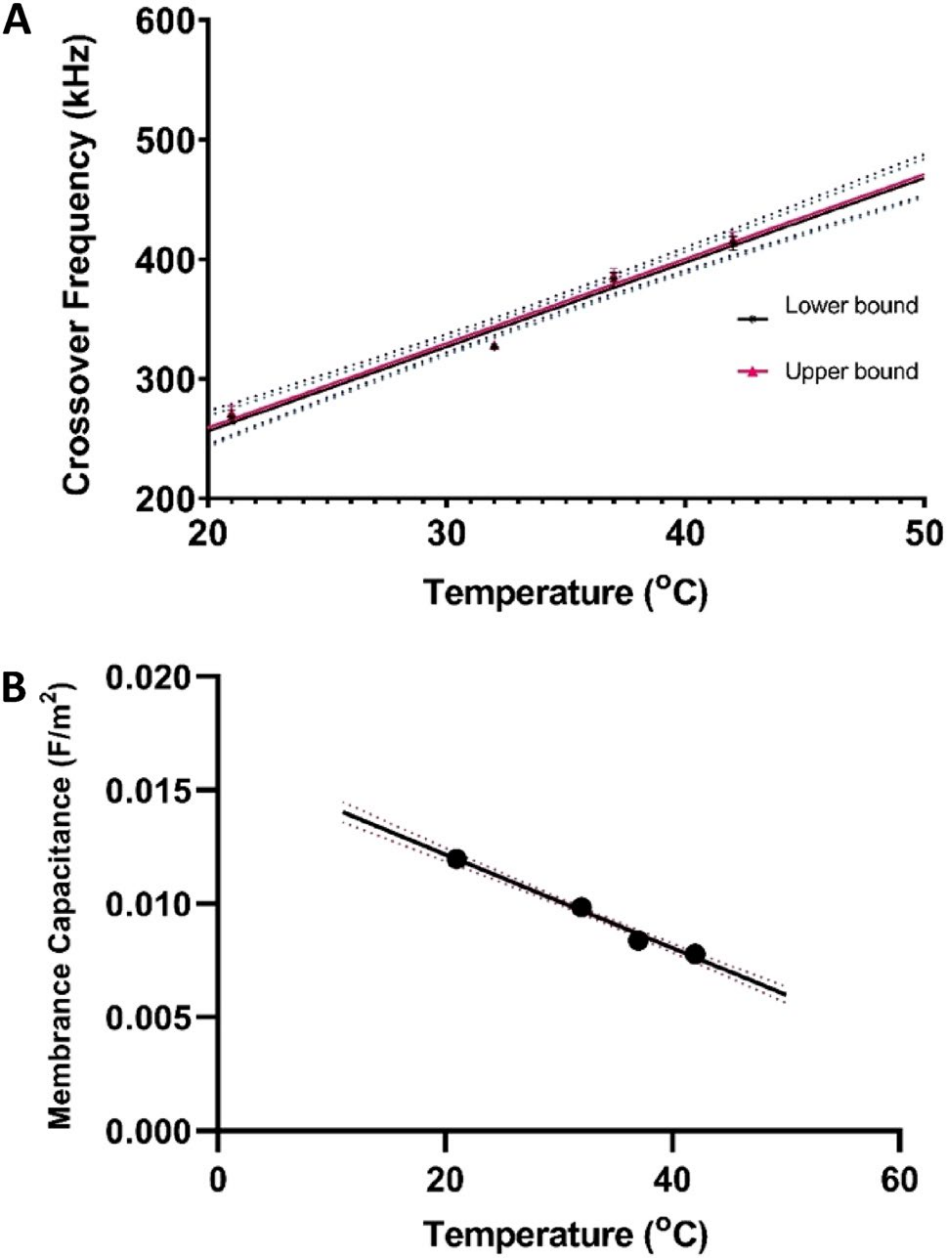
(**A**) Plots showing the best fit of crossover frequency vs. medium temperature at 0.05 S/m medium conductivity. (**B**) The experimental data were fitted with the oblate ellipsoidal single-shell model given in Eq. 8, and the cell-specific membrane capacitance is plotted against medium temperature. The data points were based on four samples (six repetitions of each sample) at each temperature value under consideration.

Temperature is a significant factor that affects the dielectric properties of biological cells. In this study, we investigated the effects of temperature on the crossover frequency and membrane capacitance of normal sheep RBCs obtained from VWR at a known medium conductivity of 0.05 S/m. Based on our preliminary work, there was no significant difference in the crossover frequency observed between bovine and sheep-derived RBCs. This study is relevant for investigating the effects of hyperthermia, an abnormally high body temperature. We examined how the plasma membrane capacitance of the cells varies with increasing temperature. Figure 9 shows the lower and upper bounds of the crossover frequency at different temperatures ranging from 21 to 42 °C. Statistical analysis indicated no significant difference between the upper and lower bound crossover frequency, as the P_value_ > 0.05. A theoretical study of cell membrane thermal capacitance response revealed that temperature elevation leads to higher potential gradients. This increase in potential gradients aligns with the classical capacitor formula, which indicates a reduction in capacitance of the extra-membranal regions^99^, as also observed in Fig. 9. An increase in temperature is predicted to result in higher membrane fluidity due to weaker intermolecular interactions^100^. Also, lower membrane fluidity is expected to reduce permeability by limiting the ability of permeant molecules to diffuse through the lipid bilayer, as suggested by the solubility diffusion model^101^.

DEP crossover frequency offers a simplified and easily interpretable parameter for characterizing cell behavior under the influence of non-electric fields. This makes it more advantageous for various practical applications when compared to other DEP spectra^35^. Similarly, the DEP crossover can be used to estimate the frequency-dependent force, as shown in Eq. 7, and other DEP properties like the dielectric permittivity, cytoplasmic conductivity, and specific membrane capacitance. A relevant study by Michael et al. determined the ranges of dielectric permittivity, cytoplasm conductivity, and specific membrane capacitance of mouse hippocampal neuronal and glial cells using DEP crossover frequency^42^. Additionally, theory predicts the reversal of the dielectrophoretic force each time the instantaneous frequency periodically traverses the crossover frequency^43^. Generally, the crossover frequency and the real part of the CM factor estimate the membrane capacitance and the intracellular properties of the cells.

### Performance comparison with an electrorotation (ROT) measurement

It is essential to inspect and compare the results obtained from our PPM device platform with other electrokinetic techniques, such as electrorotation, which was used by Gascoyne et al. to study the dielectric properties of uninfected erythrocytes obtained from human blood (group 'O')^51^. O-type RBC results agree and are consistent with the dielectric properties of the point-and-planar microwell device platform designed in this research. Gascoyne et al*.* obtained the properties of the RBCs using the modified electrorotation method^51^.

Packed O-type RBCs are suspended in the PPM device, and the DEP crossover frequency is noted. The movement of the RBCs in terms of nDEP and pDEP was recorded alongside the no-movement crossover frequency, where the time-averaged DEP force on the RBCs was zero. Each experimental run at one specific medium conductivity was completed in about a minute (~ 1 min.) since the propensity for net ion outflux from the cytoplasm to the suspending medium is enhanced with a prolonged measurement period, thus leading to significant errors in crossover frequency measurements^51^. A hypothesis is set up that there is no significant difference in dielectric properties between the modified electrorotation and the point-and-planar microwell platform in the DEP crossover frequency response results. The statistical student's paired t-test is used for analysis.

**Figure 10:**
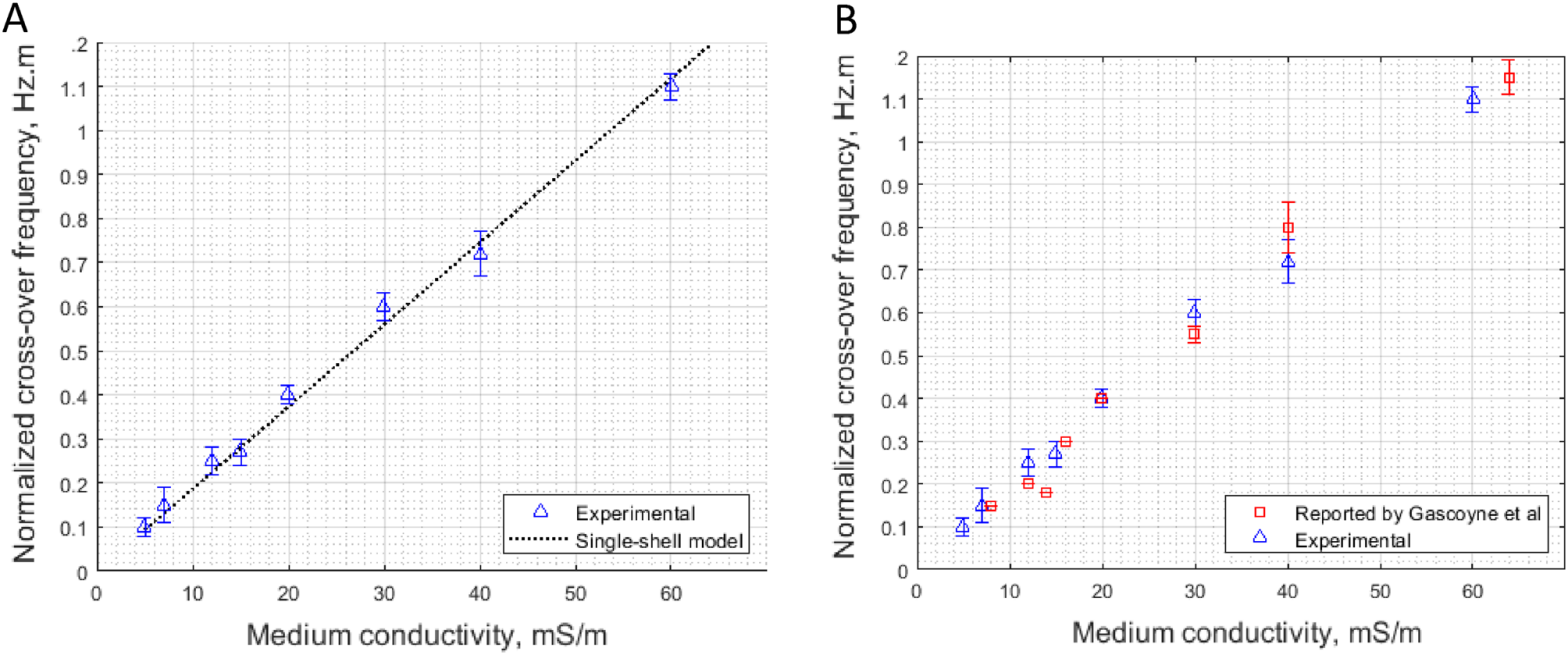
(**A**) The experimental data were fitted with the oblate ellipsoidal single-shell model given in Eq. 5. The error bars represent the measure of the variation among the six independent technical replicates. (**B**) Plots showing the crossover frequency vs. medium conductivity for the data reported by Gascoyne et al.^51^ (in red) and the data obtained through the point-and-planar microwell device (in blue). Each data set was obtained with six independent samples at varying medium conductivity. The fitted data using the PPM platform and electrorotation technique are in complete agreement.

A plot of medium conductivity *vs.* the first crossover frequency (*f*_*CO1*_) of healthy human red blood cells is obtained as described by Gascoyne et al*.*^51^, and as shown in Fig. 10. These data obtained through the point-and-planar microwell device are fitted using an optimization algorithm on Eq. 9 and normalized with the average characteristic dimension of the RBC.

9 $$r.{f}_{co1}=\frac{{A}_{0p}}{2\pi b{C}_{spmem}}{\left\{\left({\sigma }_{med}-\frac{br}{{A}_{0p}}{G}_{spmem}\right)X\left[\left(\frac{1-{A}_{0p}}{{A}_{0p}}\right){\sigma }_{m}+\frac{br}{{A}_{0p}}{G}_{spmem}\right]\right\}}^\frac{1}{2}$$

where *r* is the radius of the cell, ${{\varvec{f}}}_{{\varvec{c}}{\varvec{o}}1}$ is lower crossover frequency, A_0p_ assumes the value 1/3 for a sphere, *G*_*spmem*_ is the specific membrane conductance, and *C*_*spmem*_ is the specific membrane capacitance, ${{\varvec{\sigma}}}_{{\varvec{m}}}$ is the conductivity of the medium, where ${\sigma }_{p}= \frac{br}{\mathrm{a}}{G}_{spmem}$, and $b= \frac{c}{(2\mathrm{c}+\mathrm{r})}$. For a sphere, c = r.

It is evident from Fig. 10 that subjecting RBCs to the same preparatory conditions as outlined in the electrorotation article by Gascoyne et al*.* (8.5% (w/v) sucrose plus 0.3% (w/v) dextrose solution) provides comparable results irrespective of the method of analysis utilized. The RBCs' specific capacitance, conductance, and dimensional ratio remain consistent. Figure 10 displays a plot of $r*{f}_{co1}$ (collectively referred to as the normalized frequency, calculated using modified Eq. 9) against medium conductivity. The DEP crossover frequencies were obtained through DEP experiments with medium conductivity values ranging from 0.005 S/m to 0.06 S/m. Using a AC signal generator, the frequency was swept from 1 kHz to 50 MHz, and the crossover frequency was determined for each sample run.

The crossover frequencies increased with medium conductivity (electrorotation and PPM platform) in both cases. Additionally, the intercepts on the y-axis were positive, indicating that the dielectric properties of the RBCs and the cell's dimensions were consistent. Therefore, non-linearity in dimensions or intrinsic electrophysiological properties that could have introduced complexity into the analysis has been ruled out^90^. The paired *t* test (two-tailed) performed on the crossover frequency measurements for each conductivity, with 7 degrees of freedom and α = 0.05, comparing the experimental and Gascoyne's reported values, resulted in a t-stat value of 0.2005. This value is less than the t-critical value of 2.365. Therefore, we fail to reject the null hypothesis that no significant difference exists between the modified electrorotation and the point-and-planar microwell device crossover frequency measurements. The obtained crossover frequency response values are then used to estimate the dielectric properties of interest. The membrane's dielectric parameters obtained using the PPM device platform are compared to the reported data using electrorotation^51^ and are given in Table 1.

**Table 1:** Calculated membrane properties from the PPM device compared to the reported values from electrorotation^51^.

Specific membrane properties	Human red blood cell (group O)	
	Gascoyne et al.^51^	Microwell fit values
Capacitance $(\mathrm{mF}/{\mathrm{m}}^{2}$)	11.8	10.5 ± 1.7
Conductance ($\mathrm{S}/{\mathrm{m}}^{2}$)	271	266 ± 6.0

The estimated specific membrane capacitance and conductance shown in Table 1 indicate that our experimental results are consistent with those reported using electrorotation^52^. Based on the simulations presented in Fig. 5, different electrode shapes and spacing resulted in changes to the dielectrophoretic force (as described in Eq. 5) due to variations in electric field density. Since the DEP factor is influenced by the intrinsic properties of cell components relative to the cell's age, this can lead to a change in DEP crossover frequency, which is investigated in this study. This investigation effectively rule out any issues with variability in cell response when different shaped point electrodes are used in the PPM device platform. To verify these effects, human red blood cells obtained from Innovative Research Inc. were suspended in a 50 g/L dextrose solution. The spacing between the point and planar electrodes was adjusted only through the point electrode. Once the appropriate spacing had been established (i.e., 25, 50, 75, and 100 µm), it was kept constant by using an epoxy sealant.

### Experimental design and statistical analysis

Statistical analysis was conducted to determine the interaction effects between the two categorical variables (electrode spacing and shape) on the continuous dependent variable (crossover frequency). The results showed no statistically significant interactions (Row factor *p* value = 0.2146, Column factor *p* value = 0.8868).

The tabulated results from the experiments conducted at different electrode shapes and spacings are presented in Table 2. These results demonstrate that operating the device with an inter-electrode spacing of 25–100 µm would not change either the crossover frequency or the cell membrane properties. This observation is consistent with the literature^38,44^. Therefore, operating at electrode spacing distances (25–100 µm) does not affect the crossover frequency determination since this is the point where ${F}_{DEP}=0$. This implies that the gradient of the electric field is inconsequential at the crossover frequency point. From Table 2, the triangle-shaped electrode had the greatest accuracy compared to the other two shapes.

**Table 2:** The average crossover frequencies (kHz) of human red blood cells were determined using each electrode shape and spacing at a fixed medium conductivity of 0.052 S/m. The precision of the triangular-shaped electrode data is better than the other two designs. However, no statistically significant interactions were observed between the spacing and electrode shape when analyzing the crossover frequency data using a two-factor experimental design.

Design	Electrode spacing			
	25 µm	50 µm	75 µm	100 µm
Square	265 ± 4.82	269 ± 0.13	268 ± 2.06	266 ± 3.90
Triangle	270 ± 0.02	269 ± 0.40	269 ± 0.72	269 ± 0.72
Semicircle	269 ± 1.30	268 ± 2.60	266 ± 4.30	269 ± 0.001

## Conclusions

This article focuses on using dielectrophoretic crossover frequency as a characterization tool to address a gap in developing a cost-effective platform for disease diagnostic applications. The article introduces a novel characterization point-and-planar microwell platform (PPM), which has several advantages, including efficient inter-electrode spacing that enables real-time exploration of cell behavior at diverse spatial locations within the device, faster cellular response to electric field effects, and preservation of the bioparticle's integrity. The article also details the rationale behind the choice of material for the fabrication of the microwell platform.

The device, which operates on the principle of DEP crossover frequency, was numerically and experimentally investigated to determine the effect of electrode shape and inter-electrode spacing on DEP factor (|E.E|), a variable that determines the dielectrophoretic force of a bioparticle. This investigation narrowed the inter-electrode spacing to between 50 and 100 µm. The PPM device's validation was performed using human RBCs (group O) and compared the results to electrorotation technique.

The article also reveals relevant red blood cell properties variations based on their storage age and metabolic stress. Also, compared to an individual human baseline DEP measurement, the changes in these properties, such as conductivity and permittivity of the red blood cells, can help precisely quantify the health and age of a human blood cell suspension. This contribution is significant to the DEP world since researchers can now use this knowledge to develop a correlation factor for their cells during experimentation, depending on the storage age.

## Data Availability

The datasets generated during and/or analyzed during the current study are available from the corresponding author upon reasonable request.
